# Pursuing Synaptic Plasticity From Cortex to LTP in the Hippocampus

**DOI:** 10.1002/hipo.23665

**Published:** 2024-12-13

**Authors:** Tim Bliss

**Affiliations:** ^1^ Group Leader Emeritus The Francis Crick Institute London UK

## Abstract

Here I describe how an interest in synaptic plasticity took me from a PhD at McGill, where I worked on activity‐dependent plasticity in the responses of single units in the association cortex of anesthetized cats, to a collaboration with Terje Lømo in Per Andersen's laboratory in Oslo in 1968–9. There we followed up on Lømo's discovery of LTP, published as an abstract in 1966, to produce the first detailed description of the phenomenon. Later, in London, Tony Gardner‐Medwin and I showed that LTP lasting for days could be obtained in the awake rabbit. The two papers were published together in the *Journal of Physiology* in 1973. I relate how difficulties in replicating our results in English rabbits, and the failure of the first attempts to obtain LTP in slices of the dentate gyrus, led to my abandoning work on LTP for a few years, returning to the fray in the late 1970s through a collaboration with Graham Goddard at Dalhousie University in Halifax, Canada.

I came relatively late to an interest in neuroscience. In 1960 I was a physics undergraduate at McGill University in Montreal with almost no knowledge of biology when in my second year I saw a film about the nervous system made by the Bell Telephone Company, and learned for the first time about spinal reflexes and, according to the film, the extraordinary similarities between the brain and an advanced telephone network. I was hooked, and in my second year I switched to Physiology. One of the professors in the department, Ben Delisle Burns, had developed a preparation in which a slab of cortex was isolated from the rest of the brain using a wire tool to undercut cortical tissue without interfering with the blood supply, which was delivered from the overlying pia mater. Burns told me that he was passionately interested in the neural basis of memory, and if I wanted to do a PhD with him, that is what I had to work on. This was music to my ears. Burns accepted, and indeed took as a statement of the obvious, the celebrated rule governing synaptic plasticity proposed by another McGill professor, Donald Hebb. What was needed was proof that changes in activity could indeed cause changes in synaptic strength. So for my PhD project I recorded extracellular single unit activity in isolated slabs in the association cortex of the anesthetized cat. I set up a stimulating electrode nearby, and adjusted the strength of single test stimuli so that the recorded cell responded at a short latency spike with a certain probability, *p*. This probability defined the “conductivity” of the putative polysynaptic pathway between stimulating electrode and recording micropipette. Once a stable p had been achieved, I increased the rate of stimulation for a short time either to the test stimulating electrode (homosynaptic conditioning) or to a second stimulating electrode that was able to fire the target cell through a different pathway (heterosynaptic conditioning), and looked for a lasting change in the probability of my target cell responding to the standard test stimulus. The project was challenging, not least because of the difficulty of maintaining contact with the recorded cell for the hour or so that was minimally required to complete a conditioning experiment. Furthermore, information about the test pathway was limited to the latency and probability of the unit response, and the pathway was rarely likely to be monosynaptic. Although I obtained some evidence for activity‐depended changes in synaptic strength (Bliss, Burns, and Uttley 1968) and I remained committed to the search for synaptic plasticity in the brain, it was clear that the isolated slab preparation was not the way forward.

By a fortunate chance during my last year at McGill in 1966–7 the departmental library acquired a book that was to shape my future. Entitled The Brain and Conscious Experience, the book brought together papers presented at a meeting organized by Sir John Eccles in the Vatican in 1964. The chapter that opened my eyes to a far more promising experimental approach to the study of synaptic plasticity in the brain was an analysis by Per Andersen of the University of Oslo of responses evoked by stimulation of projections to and within the hippocampus (Andersen 1966). Thanks to the laminated organization of hippocampal neurons and their synaptic projections, the population responses evoked by stimulation of the perforant path fibers projecting monosynaptically from entorhinal cortex to dentate granule cells could easily be recorded with extracellular microelectrodes. Stimulation of the perforant path produced monosynaptically‐generated extracellular population responses which were maximally negative in the central third of the stratum oriens where perforant path fibers made en passant synaptic contact with the dendrites of granule cells, and which reversed polarity and became positive in the cell body layer. Moreover, these population synaptic response (field EPSPs) could easily be distinguished from the population spike generated by the synchronous firing of postsynaptic cells, which was superimposed on the field EPSP and was maximally negative in the cell body layer. All this organized simplicity was a million miles away from attempting to record unit responses in the tangle of cortical neuronal space. What is more, the monosynaptic perforant path projection was in the very part of the brain which I knew, from the work of McGill's Brenda Milner on the patient HM, was vital for the laying down of new episodic memories. It was clear I had to learn about field potentials.

In 1966, Burns had been appointed head of the Department of Neurophysiology and Neuropharmacology at the MRC National Institute for Medical Research in Mill Hill, London, and he invited me to join him there after I had completed my PhD. I took up my post in the fall of 1967, and set up a lab intending to record field responses from the hippocampus of anesthetized cats. For reasons that I now find hard to recall or understand I made little headway. Eventually I contacted Per Andersen and asked if I could come to his lab to master the technique. We talked about my interests and he told me “if you are interested in synaptic plasticity, you must talk to my PhD student, Terje Lømo. He has found something that I think will interest you.” I arrived in Oslo in the fall of 1968, funded by what my indulgent employers, the Medical Research Council, called a “special leave of absence”, accompanied by a Treasury approved uplift in my salary to provide for the greater cost of living in Norway (the Treasury was not to know that the student accommodation Per had arranged for me was a good deal less than the rent I was paying in London). I was soon introduced to Terje. He told me about the experiments on frequency potentiation at perforant path—granule cell synapses he had done a couple of years before showing that repeated episodes of high frequency stimulation could produce a long‐lasting potentiation of synaptic efficacy at these synapses. It was clear to both Terje and Per that this property had the potential to serve as a memory‐encoding mechanism, but neither chose to continue these experiments at the time, and only the abstract of a talk Terje had given at the annual meeting of the *Scandinavian Physiological Society* in 1966 had been published (reproduced in Lømo's paper in this issue). I was greatly excited by this revelation, and I suggested—implored might be a better word—that we should join forces to look again at synaptic plasticity in the perforant path. Terje at that time was exceptionally busy with his final experiments and writing up his thesis on the spread of excitation and inhibition in the hippocampus following local activation of the perforant path. However, he agreed to spend 1 day (and night) a week on the project. During that year in Oslo I was also working with Per and a medical student, Knut Skrede, on other projects relating to the events that generated the different components of hippocampal field potentials (Andersen, Bliss and Skrede 1971a, 1971b), by no means uninteresting, but not of the same significance as the experiments with Terje on what is now known as LTP.

In our initial experiments we exposed the hippocampus bilaterally to allow accurate placement of electrodes, and used one side as a control, giving test pulses every few seconds for the duration of the experiment, while on the other side the test pulses were interrupted by episodes of brief tetanic stimulation (usually at 15 Hz for 15 s. but sometimes at 100 Hz for a shorter time). We later changed this bilateral design when we became alarmed that the high frequency stimulation used to induce potentiation might affect the resistance or tip geometry of the tungsten‐stimulating electrode or the excitability of the axons in the vicinity of the electrode tip, resulting in an artefactual increase in strength or distribution of the stimulating current. (If we had been able to monitor the presynaptic population action potential, we would have had a measure of stimulus efficacy but in the intact animal, in contrast to hippocampal slices, the presynaptic spike is rarely seen). In his thesis, Terje had shown that perforant path fibers leaving the entorhinal cortex pass through a bottle neck in the angular bundle before fanning out to innervate the septotemporal extent of the hippocampus. Aware of this, I suggested we adopt a unilateral design in which two recording electrodes were placed along the long axis of the dentate gyrus, with a stimulating electrode placed in the angular bundle to evoke test responses from both recording sites. A second stimulating electrode was positioned close to one of the recording electrodes to provide high frequency stimulation to that pathway only. With the tetanus delivered through this separate stimulating electrode the problem of electrode polarization was resolved.

Our first experiment, on October 14, 1968, employed a bilateral design and was hugely encouraging (see below, and Figure 1). Something of the elation we experienced during that night is captured in a letter I wrote a few days later to my girlfriend in England: “Terje and I did an experiment last week, which had us yelping with excitement throughout the night”. I remember vividly the exhilaration of that night, and the sustained excitement that continued through the following months as we confirmed and extended our findings. When an experiment worked it could last for hours, and as we usually started recording late in the evening experiments often continued through the night. We had access to an instrument made by Nuclear Chicago, which could be rigged to sample and average evoked responses. There were times when we were able to leave our experiment in the hands of the averager, with the Grass camera triggered to record each response by an external pulse from a bank of Tektronix pulse generators. We would then retreat down the corridor to the departmental library to play ping‐pong on the library table. This was a time of elation and laughter, and it marked the beginning of a close friendship that has lasted till the present, as we sit at our computers writing these reminiscences in Terje's house overlooking the Oslo fjord.

**FIGURE 1:**
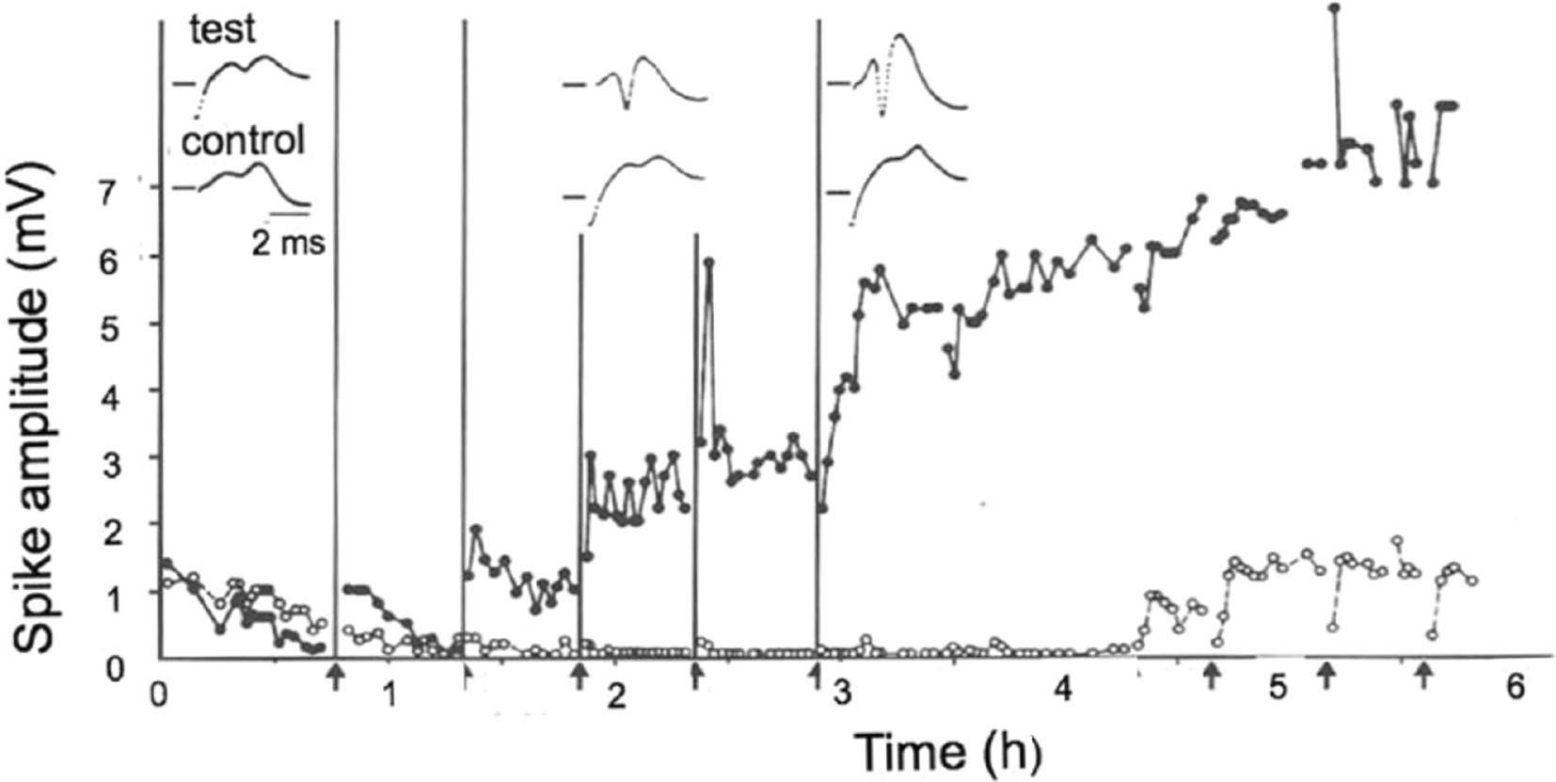
The first experiment by Bliss and Lømo in September 1968 showing a lasting potentiation of the population spike in the dentate gyrus following repeated tetanic stimulation of the perforant path (arrows, filled circles). On the control side (open circles) the population spike drifted to near zero but was revived by tetanic stimulation at the end of the experiment. This experiment was not included in Bliss and Lømo (1973) because of the downward drift of responses at the beginning of the experiment, and the upward drift of the potentiated response over the last 3 h. It was reproduced by Eccles (1970); see also Bliss and Collingridge (2019).

Toward the end of 1968 Sir John Eccles paid a visit to Per's lab in Oslo. We were convinced by then that LTP was a genuine phenomenon, but we had analyzed in full only the first experiment (all our results were recorded as the responses to single test stimuli, and the measurement and plotting of the often hundreds of responses recorded on reels of film could take several days). That experiment (see Figure 1), showed abrupt and persistent increases following each of a series of tetanic stimuli, but was marred by a steadily declining response on the control side, and for the last few hours an upwardly drifting response on the potentiated side. It was impressive but hardly publishable. Eccles became very excited when he we showed it to him and asked if he could take it away with him. Naturally, we were more than happy to present a copy to perhaps the most celebrated neuroscientist of his day—though we were hardly expecting to see our unpublishable experiment reproduced in two of his subsequent books, the first (Eccles 1970) appearing in the same year as the abstract of a talk I gave to the *Physiological Society* (Bliss and Lømo 1970), the first of our publications to refer to our work in Oslo.

By the end of my year in Oslo Terje and I were in no doubt that the phenomenon was genuine. The majority of the 20 or so experiments that we carried out showed potentiation of one or more components of the evoked response. In some experiments we saw potentiation of both components of the evoked response (field EPSP and population spike), while in others only one component was potentiated, usually the population spike (the latency of the spike was reduced or its magnitude increased or both). We used a restricted range of tetanization protocols: trains at 10–20 Hz for 10–15 s was the most common, though we also saw LTP with trains at higher frequencies (100 Hz for 3–4 s). This last result suggested that firing of granule cells during the tetanus was unlikely to be a requirement for LTP since at those higher frequencies a population spike was only evoked by the first stimulus of the train.

In the summer of 1969, we both attended a NATO sponsored summer school on synaptic transmission at Varenna, on Lake Como in Italy. Lectures were given by a number of prominent neuroscientists, and the proceedings were chaired by Bernard Katz, the Nobel Prize winning neurophysiologist who among many other major achievements had introduced the concepts of quantal analysis. On the last day, the students were invited to give a 10 min talk on their current projects. Terje had to leave early and I gave the talk on our findings. What happened next I described in a letter to Terje some years later: “Katz I remember because he gave a masterly impromptu summary of proceedings at the end of each day, and because of his “damning with faint praise” comment after my talk. Actually, it was not even faint praise, it was faint dismissal. “I do not see what is interesting about this – it has been known for years that repetitive activity will cause changes in synaptic strength at the neuromuscular junction and in the spinal cord.” I remember also not feeling in the slightest degree disheartened by this rejection—if the great BK could not see the importance of our finding that was his problem!” But Katz's skepticism was a harbinger of the lack of immediate excitement which greeted the publication of the two papers in 1973.

In September 1969, I returned to NIMR and prepared to continue experiments on LTP. Terje also moved to London with his family to take up a post‐doc position in Ricardo Miledi's lab at University College London (UCL), where he began the momentous series of experiments with Jean Rosenthal which established that the restriction of ACh receptors on muscle fibers to the motor endplate was controlled not by a trophic factor secreted by the nerve innervating the muscle but by muscle action potentials evoked by afferent nerve activity (Lømo and Rosenthal 1972). Roughly once a week Terje took the Northern Line of the London underground system up to semi‐rural Mill Hill, walked up the hill to my lab in the gaunt but imposing green‐roofed bulk of the MRC National Institute for Medical Research. Our intention was to round off our initial study of synaptic plasticity at perforant path synapses by varying the tetanus frequency and duration so we could better define the parameters of LTP‐inducing stimulation, and to work up an experimental protocol to test our indirect evidence that LTP was input‐specific (Bliss, Gardner‐Medwin, and Lømo 1973).

Things did not go well at Mill Hill. I had secured a supply of rabbits similar to the animals we had used in Oslo, and the anesthetic regime was the same as in Oslo. The evoked responses and depth profiles elicited in the dentate gyrus by stimulation of perforant path afferents were identical to the responses we had recorded in Oslo. However, we repeatedly failed to elicit LTP using a range of high‐frequency trains that had worked reasonably reliably in Oslo. Eventually we called a halt to experiments that were getting nowhere. While we were perplexed and frustrated by this failure, which we now believe must have been stress‐related (Lømo 2018), at no point did we lose faith in the experiments we had done in Oslo, and we set about writing up our results. Meanwhile I was involved in two other LTP projects. With Tony Gardner‐Medwin, also at UCL, I embarked on experiments to look at LTP in the awake rabbit. Tony had experience of recording from freely‐moving rabbits and our experiments in his lab went relatively smoothly, providing evidence that LTP could persist for a day or more, and suggesting that there was a strength threshold for the induction of LTP. It seems likely that these animals, which were allowed time to recover from the acute procedure of implanting electrodes in the hippocampus, and were handled regularly after recovery, became accustomed to the recording procedure, and thus were less stressed than the animals anesthetized for acute experiments.

Tony and I had access to a DEC PDP8 lab computer to store and analyze evoked responses, relieving us of the tedium of measuring yards of film. Tony shared the computer with Jim Pascoe, a fellow member of the Physiology Department at UCL, and one day when we were recording from one of our rabbits, unmindful that we had strayed into Jim's time for access to the computer, an early email message, preceding the general adoption of this mode of communication by several years, appeared on the monitor. It read: Hey, it's my turn. Get the f**k off my computer. Jim. Without the asterisks. We did.

Meanwhile I was collaborating with Chris Richards, who had moved to NIMR from Henry McIlwain's laboratory where he had mastered the technique, pioneered by McIlwain, of maintaining slices of cortical tissue in vitro. Chris was studying synaptic responses in slices of olfactory cortex, and in hippocampal slices cut longitudinally rather than transversely, so that almost the entire rostrocaudal extent of the lower blade of the dentate gyrus was retained. These were the same synapses that Terje and I had studied in the upper blade of the dentate gyrus in the intact animal. Chris and I found that many features of synaptic transmission that could be seen in vivo, including paired pulse facilitation, frequency potentiation, post‐tetanic potentiation and recurrent inhibition, were reproduced in the longitudinal slice preparation (Bliss and Richards 1971). But we never saw any sign of LTP. We concluded that extrahippocampal modulatory inputs—perhaps adrenergic or cholinergic—were required to allow LTP to become established, and we abandoned our experiments—perhaps the biggest mistake of my career. (We now know that to obtain LTP in the dentate gyrus, in contrast to areas CA1 or CA3, inhibitory synapses must be blocked). But Chris and I can claim some indirect credit for the in vitro revolution that was soon to come. Knut Skrede, the medical student in Per Andersen's lab with whom I had worked when I was in Oslo, visited Mill Hill for a few weeks during the summer of 1970 to write up the work we had done together in Oslo. While there he was able to watch experiments on hippocampal slices, and on his return to Oslo he introduced the preparation to Per's lab. It was Knut who had the idea of cutting transverse slices, thus opening up the full trisynaptic circuit to examination, and creating one of the most productive and enduring preparations in neuroscience (Skrede and Westgaard 1971).

In the summer of 1970, Terje and I set out to write the first of many drafts of the paper documenting our Oslo experiments. We were aware of the significance of our findings, and were anxious to produce a paper worthy of the phenomenon. The result was that we were never quite satisfied. We seem to have been unbothered by the fact that we had published an abstract of a talk I had given to the *Physiological Society* in the spring of 1970 (Bliss and Lømo 1970), and Tony and I had similarly published an abstract of our experiments on the awake animal (Bliss and Gardner‐Medwin 1971, 1973). LTP was now in the public domain but, as with Terje's abstract in *Acta Physiologica Scandinavica*, (Lømo 1966) it went almost entirely unnoticed. Still we went on refining our manuscript. During 1972, Terje and I exchanged successive typescripts by snail mail between Oslo and London. Eventually Tony Gardner‐Medwin who had quickly written up the experiments on the freely‐moving rabbit, told us he could wait no longer. So in the spring of 1973 we submitted both papers to the *Journal of Physiology*. (We are sometimes asked why we did not submit to *Nature or Science*. We never considered that an option, given the amount of data we needed to present in order to make a convincing case). Within a week I had a call from Bernard Ginsborg in Edinburgh, one of the editors of the *Journal of Physiology*. He began by congratulating me on “this interesting work.” However, he continued, “while I have no problem with the second paper, I have the impression that the first paper, the one by Bliss and Lømo, has been somewhat hurriedly written.” I said something non‐committal like “Oh, really?”, possibly with an undertone of “What??!!” while also thinking “this is going to make a great story.” Both papers were published with minimal changes.

An aside on naming the new phenomenon. Why did we call it long‐lasting potentiation? And why, after a period of nominal instability, did it finally settle down as long‐term potentiation? Terje and I had several discussions about whether we should call it long‐lasting potentiation or long‐term potentiation. I have a hazy memory of Terje informing me that long‐lasting potentiation was better English. However, what we should both have anticipated was the inevitability that an acronym would emerge—and that acronym certainly was not going to be a tongue twister like LLP. So LTP it became.

Surprisingly, and surely mistakenly, by the time the two papers had been published in 1973 I had stopped working on LTP. Discouraged by the failure to find LTP in vitro in the dentate gyrus, and by my continuing failure to obtain LTP in the anesthetized rabbit, I left the field for several years and worked on other interests (among them plasticity in the frog optic tectum, supernormal conduction velocity in tortoise olfactory nerve, and an analysis of hippocampal pathways in the reeler mutant mouse). Meanwhile, back in Oslo, Terje and Tony Gardner‐Medwin were having similar difficulties in obtaining LTP in anesthetized rabbits, as Terje describes in his memoir in this series (Lømo 2024), and eventually they too moved on to other interests.

Others were more adventurous. Graham Goddard from Dalhousie University in Halifax, Nova Scotia had read our paper and while on sabbatical in London in 1974 he asked me if LTP could be obtained in the anesthetized rat. I said “let's find out.” We did a single experiment together and again failed to see LTP. But Graham was sufficiently beguiled by the beautiful field potentials he had seen in Mill Hill that when he returned to Dalhousie he began his pioneering work on LTP in both awake and anesthetized rats.

For the next few years the running on LTP was made principally but by no means exclusively by Graham Goddard and a talented group of students (Bruce McNaughton, Carol Barnes, and Rob Douglas) in Halifax, Nova Scotia and by Gary Lynch and collaborators in Irvine California, invariably working on the rat hippocampus, either in vivo or in vitro. In 1979, I began a collaboration with Graham, then still at Dalhousie University in Halifax, on the influence of monoaminergic pathways on the induction and maintenance of LTP in anesthetized rats (Bliss, Goddard, and Riives 1983). In the early 1980s, Annette Dolphin and later Marina Lynch brought biochemical expertise to the Physiology Department at NIMR, and we began a series of experiments, at first using push‐pull perfusion of the hippocampus in anesthetized rats, to assess whether LTP at perforant path synapses was accompanied by a persistent increase in glutamate release, a question my lab continued to address using different techniques for the rest of my career, culminating in the development with Alan Fine and Nigel Emptage of optical quantal analysis, using confocal microscopy and intracellular Ca^2+^‐sensitive dyes to measure quantal parameters at individual Schaffer collateral/ CA1 synapses. This is still the only technique that directly addresses the locus of LTP at single synapses in organized hippocampal tissue. Here the evidence has consistently indicated that presynaptic mechanisms contribute to the expression of LTP. The induction process at most hippocampal synapses requires activation of postsynaptic NMDA receptors, leading to an increase in the surface density of postsynaptic AMPA receptors. After more than 50 years the expression mechanisms of LTP and its involvement in learning (Morris 2024) are still incompletely understood. However, interest in the phenomenon first studied in a small lab in the old university buildings in downtown Oslo continues unabated, with growing evidence that defects in the molecular mechanisms that support LTP may contribute to a number of neuropathological conditions (Bliss et al. 2024).

## Data Availability

The author has nothing to report.
